# Dynamic analysis of the determinants of long-term microfinance interest rates: Macro and micro factors

**DOI:** 10.3389/fpsyg.2022.1008002

**Published:** 2022-11-30

**Authors:** Fawad Rauf, Wang Wanqiu, Li Jing, Syed Usman Qadri, Khwaja Naveed, Shams U. Rahman

**Affiliations:** ^1^College of Economics and Management, Beijing University of Technology, Beijing, China; ^2^LinKong School of Economics and Management, Beijing Institute of Economics and Management, Beijing, China; ^3^School of Management, Jiangsu University, Zhenjiang, China; ^4^Riphah School of Leadership, Faculty of Management Sciences, Riphah International University, Islamabad, Pakistan; ^5^Faculty of Management, University of Agriculture, Peshawar, Pakistan

**Keywords:** interest rate (E43), microfinance, GMM, dynamic analysis, macro and micro

## Abstract

**Introduction:**

A recent increase in interest rates has raised doubts about the stability of micro-finance institutions (MFI) A recent increase in interest rates has raised doubts about the stability of MFI in many countries. This has compelled governments to consider some MFI practices unethical.

**Methods:**

This paper studies the MFI interest rates by using a dynamic panel method to identify the determining factors of the viability, financial, and social execution of microfinance firms. The research shows that the long-term interest rate evolution depends on the anticipation of loan loss rates (LLR), profit, or macroeconomic factors like inflation and the short-term current interest rate. The Study used database of 897 microfinance institutions in 106 countries and six geographic regions with a representative sample size of 5,075 observations between 2008 and 2020. The external factors considered are the market structure (Competition), economics (inflation), cultural and technological political conditions, and banking regulations in effect (regulation). Financial costs, operational costs, the write-off rate, and the average size of the loan are the most important determinant factors in MFI interest rate fluctuations.

**Results:**

The research find that other factors like gender, legal status, and regulations also contribute to the MFI interest rate variation. The research also discovered that there is a threshold effect in the relationship between women borrowers (WB) and the interest rate. Another important finding of this study is that MFIs do not anticipate inflation in the definition of the interest rate.

**Discussion:**

From an institutional point of view, it is necessary to promote competition, as the study shows that well-regulated competition helps to keep interest rates at a reasonable level.

## Introduction

Micro-finance institutions (MFI) interest rates have fluctuated significantly in recent years. Most MFI data specifies that financial and operational costs must be offset by the increase in interest rates. Most microfinance institutions have succeeded to develop and create sustainable activities by applying interest rates that cover their overall operating costs (Rosenberg et al., 2009; Gupta and Mirchandani, 2019). The interest rate variation comes out of several factors that are less manageable by micro-credits (Nwachukwu et al., 2018). There are also micro-credits whose activities are only motivated by profit-seeking. The recent increase in interest rates has raised some questions about the stability of MFIs in many countries. This has compelled some governments to consider some MFI practices as unethical and against the best interest of borrowers, hence the capped rate of interest in some countries to fight against distortion practices is overviewed (Helms and Reille, 2004; Al-Azzam, 2016; Chikalipah, 2017). Measures have been taken in some countries, like Nicaragua, where President Ortega encouraged borrowers to default on loans (Jiang et al., 2019).

This MFI interest rate increase has been observed in most countries over the past decade and has motivated field research (Rosenberg et al., 2009; Dorfleitner et al., 2013; Gupta and Mirchandani, 2019). The primary motivation behind this research is to identify the determining factors of the viability, financial, and social execution of microfinance firms. Previous researchers failed to understand the interest rate fluctuations that stem from the anticipation of some variables like loan loss rates (LLR), profit, or macroeconomic factors like inflation. They were constructed on the estimations of static econometric models. These authors assume that the interest rate evolution observed at the (t-1) period is identical to that observed at the (t) period. However, these static analyses do not provide sufficient explications for present and future interest rates of MFI fluctuations because adjustments to changes in the financial environment are never instantaneous (Chao et al., 2019; Jiang et al., 2019).

In addition to these limitations, these works have not highlighted the MFI legal status effects and the impact of female borrowers on interest rate fluctuations (Nwachukwu et al., 2018). This article removes these ambiguities by using the Fisher hypothesis test and by showing the threshold at which the share of women in microfinance institutions has a fluctuating impact on the rate of interest.

Our study considers a different analysis of the above-mentioned literature. By using a dynamic approach, the main question that we ask in this paper is whether the increase in interest rates is bound to the anticipation phenomena. That is a system that is not spot dependent on its properties but rather depends upon the progression over time. It is important to distinguish between the current and long-term effects of explanatory variables on MFI interest rate changes (Hashemkhani Zolfani and Bahrami, 2014; Chao et al., 2019).

Firstly, the study uses the effect of the dynamics of short- and long-term by combining lagged and spot variables to show whether the value of the interest rate in periods depends on the anticipation of its value in (t-1) and the evolution trend of other independent variables. The coefficients associated with the lagged variables represent the anticipation coefficients. If the coefficient is positive and significant, this means that the trend will continue and that the MFI anticipates an interest rate increase; otherwise, it anticipates an interest rate decrease. If it is equal to zero, the MFIs anticipate that the interest rate evolution at the (t-1) period will be identical to that observed at the (t) period, which refers to the static models. Secondly, it shows that using a dynamic analysis method provides more and better information on MFI interest rate fluctuations.

Finally, the research confirms the difference between acclaimed specificities as per the legal status of microfinance establishments and their outcomes as depicted by the observation that NGOs and rural banks do not often meet expectations of solidarity practices.

The study used a representative sample of 897 MFIs over 12 years period (2008–2020). The results showed the determination of interest rates according to short and long periods.

The remaining article is structured in four sections. In section “Literature Review,” researchers present the literature review, and section “Data Description and Methodology” discusses the methodological approach. The balanced panel data results are presented in section “Results and Findings” by employing the GMM method and finally, the conclusion with the research perspectives is presented in section “Findings.”

## Literature review

Recent theoretical developments have shown that the interest rate of MFIs depends on several characteristics. These include market structure, type of clients, MFI legal status, internal, and external factors, and macroeconomic factors.

However, as with classic banks, the relationship between these factors and the MFI’s interest rate has been embryonically discussed in the literature (Dorfleitner et al., 2013; Catalán-Herrera et al., 2019). This literature focused mainly on determinants explaining the profitability of microfinance firms without considering the interest rate evolution (Cuéllar-Fernández et al., 2016) examined the determinants of MFI financial viability by using a MIX database (Microfinance Information Exchange) for the year between 1992 and 2002. Researchers identified that rates of interest and refinancing prices impact the financial viability of microfinance firms. Their results might be reconsidered, as these authors do not justify how the interest rate affects MFI’s financial performance. Gonzalez and McAleer (2011) and Rosenberg et al. (2013) argue that while MFIs have higher yield rates than classic banks, rent-seeking is not a determinant element of these interest rates (Gonzalez and McAleer, 2011; Rosenberg et al., 2013; Mimouni, 2017). Despite the resonance of their discussion, these researchers could not test their results due to the lack of evidence of the explanatory variables and the econometric method (Jiang et al., 2019).

Another empirical study uses data on subsidies D’Espallier et al. (2017) to explain the interest rate evolution. The uncertainty of subsidies makes the MFI objectives difficult to reach and causes the drifting of their social mission, and consequently interest rate adjustment increases (Hashemkhani Zolfani and Bahrami, 2014; Chikalipah, 2017; D’Espallier et al., 2017).

Relating to the MFI objectives, some authors argue that micro-finance is unable to generate high profits because they still support high costs by generating low incomes (Mersland and Strøm, 2012; Mimouni, 2017).

Furthermore, Ahlin et al. (2011) highlight the national context effect, especially macroeconomic and macro-institutional characteristics to explain the MFI’s financial performance. They showed that the interest rate, and the operating costs, could be reduced by the competition. Cotler and Almazan (2013) distinguish a positive connection between monetary expenses and the microcredit loan fee and a negative connection between the proficiency level of MFIs and financing cost (Cotler and Almazan, 2013; Janda et al., 2014; Mimouni, 2017).

Despite this extensive literature, some of these results are weak and the question of the MFI interest rate anticipation setting remains unanswered. In other words, anticipation phenomena have never been studied in the microfinance literature. Another contribution of this article is the evidence regarding the existence of a threshold effect between women borrowers and interest rates. Most of the authors who studied the effect of female borrowers on interest rates estimated that an MFI female clientele increases because interest rates increase (Dorfleitner et al., 2013). Our study showed that this relationship is not linear and from a certain threshold, about 68% of the positive impact of women on the interest rate becomes negative (Kou et al., 2019).

## Data description and methodology

To further this research on the microfinance interest rate determinants, the study used a database from the MIX (microfinance information exchange) to collect all information on microfinance institutions across the world to better ease exchange between the different MFIs. Created in 2007, the MIX aims to foster a microfinance market, allow a comparison between MFIs, and provide performance monitoring tools and data collection services. It allows to easily access to monetary and social execution data for more than 2,000 microfinance institutions worldwide, that cover 0.092 billion borrowers. MIX is earmarked for financial inclusion and transparency in the microfinance sector.

### Sampling method

For this study used a database containing 897 microfinance institutions over 12 years (2008–2020). This allowed us to take into account all the factors that can influence the interest rate economically, socially, historically, or geographically. The implementation of this sample results from a multi-step adjustment. In the first sampling phase, considering that MIX distinguishes the social and financial performance based on diamond classification on a scale of 1–5, we only used MFIs that had reached 3 diamonds. This is to have complete external reporting (financial audit).

In the second phase, the researchers removed from the sample those MFIs with portfolio values negative or less than USD 20,000 and those with operating costs greater than 350%. In the final phase, MFIs that have recorded missing data or have not reported their data for those periods have also been removed from the sample.

### Dependent variable

The research used the real interest rate (TX REAL) as a dependent variable at the expense of the real yield rate to explain the interest rate evolution. MIX does not explicitly provide this interest rate for various reasons, more than often related to confidentiality, to ensure transparency between borrowers and microcredit providers. It is also bound to the high diversity of applied interest rates and fixed banking fees. This makes it difficult to assign a uniform interest rate for each MFI. In this way, the interest rate considered in this paper derives from the nominal interest rate.


$$N\,o\,m\,i\,n\,a\,l\,i\,n\,t\,e\,r\,e\,s\,t\,r\,a\,t\,e=\frac{\mathrm{Incomes}\,\mathrm{Interests}}{\begin{array}{c} \mathrm{Average}\,\mathrm{Loan}\,\mathrm{Portfolios}\\ \left(\,1-\mathrm{Loans}\,\mathrm{lost}\,\mathrm{rate}\right) \end{array}}$$

The inclusion of loan Lost springs from the fact that when the customers stop repaying their debt, the nominal yield (real interest paid) is on a slightly downward trend compared to the facial interest rate (the total interest he would have paid if he continued to repay his debt).

The real interest rate can be calculated in two different ways:


Simple⁢method⁢(1):T⁢X⁢R⁢E⁢E⁢L=T⁢X⁢N⁢O⁢M-I⁢N⁢F⁢L

$$\mathrm{Precise}\,\mathrm{Method}\,\left(2\right):T\,X\,R\,E\,A\,L=\frac{\left(1+N\,I\,R\right)}{\left(1+I\,N\,F\,L\right)}-1$$

In this paper, the study use the second method which provides more information on the real interest rate.

### Description and variables operationalization

For this study, we have selected a set of variables considered essential. Some of these variables have been highlighted by other authors like Rosenberg et al. (2013) but adopting a different methodology from ours. The definition of variables is explained in Table 1.

**TABLE 1:** Variables.

Variables identity	Measures	Definition
Real interest rate	RIR	$T\,X\,R\,E\,A\,L=\frac{\left(1+N\,I\,R\right)}{\left(1+I\,N\,F\,L\right)}-1$ This ratio represents the total income of cash flow generated by the loans plus the fee and commissions and the income of the obligatory deposits then inflation is deducted (source: Mix^a^)
Nominal interest rate	NIR	TXNOM $=\frac{\mathrm{Interest}\,\mathrm{incomes}}{\mathrm{Gross}\,\mathrm{Loan}\,\mathrm{Portfolios}\,\left(1-\mathrm{Loan}\,\mathrm{lost}\,\mathrm{rate}\right)}$ This ratio represents all the income coming from the granted loans taking into account the obligatory deposits (Source: Mix)
Operating cost rate	OC	Operating cost/Gross loan Portfolios. It represents the costs needed to provide credit services.
Financial cost rate	FC	Equity access (Financial expenditures)/Gross loan portfolios (Source: Mix).
Loan lost rate	LLR	Difference between collected loans and loan lost/Gross portfolio of loans
Female borrowers	WB	Female borrowers/total clients in MFIs. (Source: Mix)
Average gross loan portfolios	ALP	All receivables are held by an institution from its members or clients. (Source: MIX). The Currency unit is the American dollar (USD).
Average loan size	ALS	The ratio between loan portfolios/active borrowers. (Source: MIX). The Currency unit is the American dollar (USD).
Average deposits	AD	It represents the ratio between the funds, other than contributions and mandatory contributions, collected by the MFIs from its members or clients with the right to dispose of them in the course of its activity and the total loan portfolio. Calculated from MIX data. The Currency unit is the American dollar.
Average deposits	AD	It represents the ratio between the funds, other than contributions and mandatory contributions, collected by the MFIs from its members or clients with the right to dispose of them in the course of its activity and the total loan portfolio. Calculated from MIX data. The Currency unit is the American dollar.
Gini per capita	GINI/CAPITA	Per capita income is an economic Indicator of wealth relative to each country.
Profit rate	PR	Marginal Profit × Financial income/gross Loan portfolios = Net Profit/Gross Loan Portfolio = Capital/Gross portfolio of loans. (Source: Mix)
Portfolio risk at 30 days	PAR-30	Outstanding Loan of which repayment term is > 30 days/Gross loan Portfolios. This ratio measures the quality of the portfolios which represents the part of the credit portfolio debased by unpaid credits and thus presenting a significant risk of non-repayment. Standard < 5%
Legal status	BANK CU RB NGO FINB	Binary Variable, which takes 1 if MFI Bank, and 0 otherwise. Binary determinant which keeps 1 whether the MFI is a CU, 0 otherwise Binary Variable which takes 1 if the MFI is an RB, 0 otherwise Binary Variable, which takes 1 whereas MFI represents an NGO, 0 otherwise. Binary factor, which takes 1 whether MFI is a FINB, 0 otherwise.

^a^Microfinance Information Exchange.

The first independent variable represents the overhead costs measured in terms of the ratio approached by MIX as the operating costs (OC). This variable allows us to understand the impact of administrative costs, personnel costs, and depreciation costs on MFI interest rates. According to a study conducted by Rosenberg et al. (2013), overhead costs fluctuate between 10 and 25% and represent the most decisive element in the interest rate setting (62%). The second independent variable represents the loan lost rates (LLR) measured as losses on loans that have been recorded after each accounting balance sheet. he third variable represents the financial costs (FC) or refinancing costs from donors, banks, or other MFIs.

In recent years, financial costs have continued to increase due to microfinance institutions’ growth and a decrease in potential borrowers. This has led the MFIs to turn to commercial lenders that apply higher interest rates. furthermore, another variable influencing the interest rate is the percentage of women using MFIs. Women borrowers (WB) are a very active part of the microcredit market and are therefore an appropriate target for MFIs. It is estimated that 70% of the poor and 85% of the poorest clients receiving microfinance services are women, which represents a significant and growing potential of the informal economy. Commercially, many types of research have demonstrated that the loan recovery rate observed in the female borrower population is higher than other borrowers. in this study we also control for the legal environment (whether the MFI is regulated or not), the legal status of NGO, non-banking financial institutions (NBFI), credit associations banks, and cooperatives, or whether it is a for-profit MFI or not.

### Model specification

We examine the importance of all the above-mentioned factors using a dynamic panel regression analysis. We recall that all available studies on the determinants of MFI interest rates are too often limited to a static approach, as is the case with the work of Dorfleitner et al. (2013). The studies, so far recalled, allow the correction of heteroscedasticity (method of generalized least squares) and/or serial autocorrelation of residues. These studies do not consider the intertemporal variations of the interest rates. The purpose of this paper is to correct the shortcomings noted in the static model by using a dynamic model. This latter represents a model in which one or more lags of the independent variables. This results in the paper that the interest rate in the “t” period depends on the one in the (t-1) period and the other explanatory variables that compose it.

### The estimation method

Our model is based on the one developed by Bond (2002):


(1) Y=i⁢tαY+i⁢t-1βX1+1⁢itβX2+2⁢itμ+iγ+tεi⁢t

Equation (1) is a dynamic model characterized by the presence of one or more lags of the endogenous variable, which appear as an explanatory variable. To simplify the model, the variable Y_*it*_ is studied with only one lag.

The i and t indexes respectively denote the time dimension cross-sectional panel data.

Y_*it*_ is the interest rate (presented in the form of MFI i proportion) in t period; Y_*it*_-1, the interest rate at t-1 period; X_1i*t*_ is the internal factors matrix while X_2i*t*_ is the external factors matrix. μ_*i*_, is the specific effect of i on MFI; time effect, and the error term. All the statistics relating to the error term are standardized forms and are distributed according to the descriptive statistics (having zero mean and variance equal to).

The statistical assumptions of equation are as follows (Greene, 2010). The foremost assumption is that the model is linear in parameters. The second assumption is E (ε_*it*_| x_*i*1_, x_*i*2_, …, x_*iT*_) = 0 ∀ *i*. The third assumption is var(ε_*it*_| x_*i*1_, x_*i*2_, …, x_*iT*_) = σ_ε_^2^. The fourth assumption is cov(ε_*it*_| x_*i*1_, x_*i*2_, …, x_*iT*_) = 0 given that *i* ≠ *j* or *t* ≠ *s* and the last assumption is X is a (NT × K) full column rank matrix.

It ranges widely of specification errors. Mostly, these effects are caught by the existence of individual and temporal effects, but some are only caught by the standard error term or idiosyncratic error.

If all the explanatory variables (internal and external factors) other than Yit-1 are grouped in only vector that will be noted “X,” Equation (1) can be rewritten as:


(2) $$Y_{i\,t}=Y_{i\,t-1}+\delta +\left(\alpha -1\right)\,Y_{i\,t-1}+\beta_{i}\,X_{i\,t}+\mu_{i}+\Upsilon_{t}+\varepsilon_{i\,t}$$

error term and lagged endogenous factor correlated with each other, or the potential correlation between the Xit and past events, the estimation of this Equation (2) by ordinary least squares or the fixed effect (within) give biased results. The main motivations for applying the GMM methodology can be found in Blundell and Bond’s (2000) or Arellano and Honoré’s (2001) research which generalizes this method by applying it to the investment rate of firms and the production function. According to this, we will retain two types of estimates based on GMMs (Generalized method of the moment):

Arellano and Bond (1991) built up a model to estimate the consistency in differentiating the econometric model and instrumenting term by the set of lagged values. It takes into account a possible bias of omitted variables from the specific effects (Arellano and Bond, 1991). Thus Equation (2) becomes:


(3) Δy=i⁢tαΔy-i⁢t1+βiΔX+i⁢tΔεi⁢t

Δ represents the first difference operator.

This method affords the advantage of assessing the autocorrelation errors caused by the first differentiation and removes the variations among the countries. It is convergent when the number of observations tends to infinity and the period is fixed. However, the properties of this estimator are weak when variables are highly persistent and error terms are correlated with the dependent lagged variable: $\left(Y_{i\,t-1}-Y_{i\,t-2}\right)$:

This implies that the lagged variables in level are weakly correlated with the first difference equations (weak instruments). When explanatory variables and the dependent variable are highly persistent, Bond (2002) presented to employing the estimator of the “GMM in Difference” is weak and that this estimator is not appropriate (Bond, 2002).

This model fills the gap detected in the “GMM in Difference” (weak instruments). This method combines the first difference Equation (3) and the level Equation (1). These authors found that GMM estimated efficiency.

The latter provides biased results in limited examples when the instruments have less capacity to measure. Two tests are associated with the dynamic panel GMM estimator: the Sargan/Hansen over identifying test allows us to test the legitimacy of the instruments, which tests where the null hypothesis is no correlation of the error of differential equation.

To choose between these two estimators, Bond (2002) proposes to estimate a first-order autoregression model for each variable to measure its “persistence.” If for some variables the autoregression coefficient is close to one, the Blundell and Bond (2000)’s GMM system estimator is better, otherwise, the GMM difference estimator is adopted (Bond, 2002).

In this paper, we use the “GMM system” (Sys-GMM) which has the advantage of taking into account both the difference in inter-countries and intra-microcredits characteristics.

## Results and findings

In this section, we present the different estimates to analyze whether the explanatory variables have a significant impact on the MFI’s interest rate fluctuations or whether these interest rates in defined according to the anticipations of some variables. This requires a development of the work of Dorfleitner et al. (2013) and Rosenberg et al. (2013) on the variables they had used and to extend the study in a general framework taking into account the inter-temporal variabilities of some variables. We also highlight the stationarity and cointegration tests between variables.

### Unit root test (KPSS test)

Table 2 presents the unit root test obtained with KPSS. In this test, under the null hypothesis, the variables in level are stationary or integrated with zero-order [I (0)]. This is done by including the constant and the trend. The results of the level test show that the t-statistics of all variables are above the critical values at the 1% threshold. This involves rejecting the null Ho hypothesis which assumes that data is a unit root. However, by expressing the variables as the first difference, we remark that they are all significant at 1%. Therefore, the results show that the data is stationary.

**TABLE 2:** Table of the unit root test.

Variables	Level test		First difference test		
		
	*t*-statistic	Critic value	*t*-statistic	Critic value	Result
NIR	0.7163	0.2160	0.0819***	0.2160	I (1)
FC	0.4175	0.2160	0.0288***	0.2160	I (1)
OC	0.4629	0.2160	0.0412***	0.2160	I (1)
LogALS	1.6529	0.2160	0.0373***	0.2160	I (1)
LogGINI/CAPITA	1.8166	0.2160	0.0181***	0.2160	I (1)
AD	0.4853	0.2160	0.0304***	0.2160	I (1)
LLR	0.0549	0.2160	0.1458***	0.2160	I (1)
PAR_30	0.1899	0.2160	0.0372***	0.2160	I (1)
PR	0.4846	0.2160	0.0927***	0.2160	I (1)
COMP	0.3342	0.2160	0.0125***	0.2160	I (1)
INFL	1.3279	0.2160	0.0435***	0.2160	I (1)
REG	0.9400	0.2160	0.1142***	0.2160	I (1)
ROR	0.8814	0.2160	0.0433***	0.2160	I (1)

Characteristic value is equal to 0.2160.***Means that the variable is significant at 1%. The KPSS Test Hypothesis is as follows:

H0: The Data is a unit root.

H1: The Data is not a unit root.

### Descriptive analysis of the statistical data

Regarding the developed theory, data was collected, and different variables have been highlighted. The descriptive statistics of this study are summarized in Tables 3, 4.

**TABLE 3:** Frequency of categorical variables.

Years												
2008	2009	2010	2011	2012	2013	2014	2015	2016	2017	2018	2019	2020
99	186	261	390	392	496	547	602	602	464	409	271	370

**Legal status**												

BANQ	UC	BR	IFNB	ONG	AUTRES							
517	538	100	2022	1,872	26							

**Profit status**												

Non-profit	Profit											
2855	2220											

**Regulamentation**												

Régulate	Non-regulate											
1,852	3,223											

**TABLE 4:** Descriptive statistic of quantitative factors.

	Mean	Median	Maximum	Minimum	Std. Dev.	Prob	Obs
FC	0.069807	0.064400	0.465200	0.000100	0.045818	0.000000	5,075
OC	0.245812	0.187000	2.425000	0.002000	0.199970	0.000000	5,075
AD	0.239694	0.000000	3.146000	0.000000	0.381271	0.000000	5,075
WB	0.661387	0.648400	1.000000	0.000000	0.258203	0.000000	5,075
INFL	0.069855	0.066000	1.051000	–1.074000	0.242505	0.000000	5,075
LLR	0.014993	0.004200	0.690600	–0.294200	0.036917	0.000000	5,075
AD	0.018411	0.006500	0.659900	–0.024400	0.035829	0.000000	5,075
ALP	46.959960	7284221	4.9320.9	117262.0	1.672.08	0.000000	5,075
GINI/CAPITA	3042.786	2063.071	15227.63	119.3059	2762.342	0.000000	5,075
PAR_30	0.053343	0.031800	0.737400	0.000000	0.074915	0.000000	5,075
PR	0.021588	0.035000	1.593000	–0.800000	0.140102	0.000000	5,075
ALS	1140.944	564.6000	15966.49	21.48000	1596.620	0.000000	5,075
NIR	0.436350	0.398000	1.397000	0.008000	0.191324	0.000000	5,075
RIR	0.366544	0.324000	1.355000	0.000000	0.177525	0.000000	5,075
YIELDNOM	0.330782	0.293000	1.077300	0.000000	0.160175	0.000000	5,075
YIELDREAL	0.244928	0.212400	1.016800	–0.224000	0.159230	0.000000	5,075
COMP	43.92050	41.71454	95.20945	1.000000	27.40344	0.000000	5,075

The real interest rate applied by the MFIs in the world is estimated at 36.6%. The standard deviation which reflects the dispersion around the mean was estimated at 17.75%. This result shows that the data used for the real interest rate variable are weakly dispersed. Operating costs (OC) represent the variable with the most weight on MFI costs with an average of 24.58%. Thus, MFI’s share of financial costs (FC) averaged over 7% of total costs. The results of this study also confirm that women are essential actors in microfinance. They represent a 66.13% average among borrowers. This approximation is similar to that found in the models developed by Dorfleitner et al. (2013).

The MFIs in our sample are mainly composed of non-financial institutions, NGOs are mostly non-profit (2,855) and not regulated. The average risk portfolio quality (PAR_30) is 5%, which is above the 1% threshold. Before we further this issue, it is necessary to focus on the correlation between the different quantitative variables implemented in our sample to recognize the causal connection between the factors.

### Correlation analysis test

According to Mersland and Strøm (2010), only the correlated variables of 0.8 level can bias the estimation results by generating a multi-collinearity effect (Mersland and Strøm, 2010). Thus, highlighting in the same model of LLR and provisions can bias the results because these two variables are considerably correlated at a 93% level. Interest rates (real and nominal) are also correlated with yields (real and nominal) with correlation coefficients above 80%.

According to the correlation (Table 5), NIR and DIOC is a highly optimistic relationship with each other. Interest rates are negatively correlated with competition level (COMP), average deposits (AD), average loans portfolios (ALP), and average loan size (ALS) with respective coefficients of –10%, –0.9%, –6%, 9%, –24%. It shows that there is no problem with multicollinearity.

**TABLE 5:** Results of correlation coefficients.

	PAR-30		PN		ALP	NOIR	RIR		LLR
PAR_30	1							
PR	0.001225		1						
ALP	–0.01103		0.026588		1				
NOIR	0.006255		0.072778		–0.25489	1			
RIR	0.007379		0.050536		–0.233821	0.972511	1		
PERTES/CREA	–0.008024		–0.25302		–0.043174	0.343621	0.321527		1

	**COMP**	**AD**	**D1OC**	**FC**	**WB**	**ALP**	**GINI/CAPITA**	**INFL**	**LLR**

COMP	1								
AD	–0.00673	1							
OC	0.019651	–0.0553	1						
FC	–0.01417	0.03887	–0.0421	1					
WB	0.001377	–0.1944	0.1037	0.03227	1				
ALP	–0.00638	0.17216	–0.1477	0.02616	–0.0594	1			
GINI/CAPITA	–0.01614	–0.1093	0.14348	0.05582	–0.1598	0.03371	1		
INFL	0.012289	–0.0191	0.01181	0.18229	0.04812	0.00594	–0.226454	1	
PPROV/pertes	–0.00219	–0.0337	0.22274	0.02589	–0.0195	0.00054	0.125836	–0.0625	1
PAR_30	0.002308	0.00333	0.00409	0.00657	–0.0108	–0.0018	–0.00321	0.0145	–0.0113
PR	–0.03193	–0.031	–0.5413	–0.071	0.03018	0.05199	0.043718	–0.0331	–0.2306
ALS	–0.01095	0.17378	–0.2428	0.00958	–0.5147	0.15127	0.313086	–0.0932	–0.0531
NOIR	–0.00695	–0.0751	0.69624	0.19544	0.13102	–0.1107	0.243812	0.02696	0.32645
NIR	–0.00982	–0.0604	0.63479	0.41842	0.12904	–0.0963	0.239011	0.06824	0.30849
LLR	–0.00309	–0.0386	0.26541	0.01379	–0.0531	–0.003	0.149953	–0.0815	0.93312

### Multicollinearity test

The multicollinearity test is calculated by the Variance Inflation Factor (VIF) test that is given below in Table 5. In this analysis, the ratio is calculated for each explanatory variable. A high VIF value indicates the sign of multicollinearity in the model, normally the value is greater than 5. Table 6 shows that VIF values of independent variables are less than 5. So, there is no multicollinearity in the model.

**TABLE 6:** VIF test.

Variable	VIF	1/VIF
FC	1.17	0.854308
OC	1.16	0.858525
AD	1.13	0.885683
WB	1.12	0.890344
INFL	1.09	0.913906
Mean VIF	1.14	

### Heteroscedasticity test

The Heteroskedasticity test is calculated by the Bruesch Pagan LM test which is given below in Table 7. The null hypothesis is the constant variance that is not rejected because the p-value is greater than 5%. So variance is constant and there is no heteroskedasticity in the model.

**TABLE 7:** Breusch-Pagan/Cook-Weisberg test for heteroskedasticity.

Number of instruments = 165		Wald chi^2^(6) = 131.01
Prob > chi^2^ = 0.0000		
Ho:	Constant variance	
Variables:	Fitted values of hc	
chi^2^(1)	2.23	
Prob > chi^2^	0.1352	

### Ramsey RESET test

The Ramsey RESET test is used to check model misspecification. It is given below in Table 8. The null hypothesis is model has no omitted variables that are not rejected because the p-value is greater than 5%. So the model has no omitted variables and there is no misspecification in the model.

**TABLE 8:** Ramsey RESET test.

Ramsey RESET test using powers of the fitted values	
Ho: model has no omitted variables	
F(3, 177) =	1.19
Prob > F =	0.3149

This test identified the overidentifying restrictions in the model.

H0: overidentifying restrictions are valid.

The results indicate that overidentifying restrictions are not valid in the model.

## Findings

In Table 9, we have pointed out five estimation models that combine the lagged of the explanatory variable, some level variables, and the differential factors in order 1. The first model represents the reference model, which considers all variables except interactions. The objective is to analyze the individual impact of each variable on the interest rate change.

**TABLE 9:** Estimation results from GMM-system.

	Variables	Model I RIR	Model II RIR	Model III RIR	Model IV RIR	Model V RIR
	RIR(–1)	0.340156***	0.345071***	0.300758***	0.295242***	0.298650***
Internal factors	FC	1.215206***	1.203386***	1.421381***	1.383806***	1.368810***
	ΔFC	0.655167***	0.675491***	0.662583***	0.686853***	0.734953***
	OC	0.473618***	0.442673***	0.654095***	0.124968*	0.115015*
	ΔOC	0.032362*	0.036070**	0.059106***	0.066563***	0.072832***
	PR	–0.012391**	–0.012210**	–0.051068***	–0.059156***	–0.054855***
	PR (–1)	–0.003895	0.530712***	–0.015721**	–0.028360***	–0.027342***
	WOR	0.405721***	0.530610***	0.482356***	0.451211***	0.585807***
	ΔWOR	0.268899***	0.140121**	0.101881	0.064654	0.585807**
	LLR	0.003872	0.130143**	0.005896**	0.007307**	0.007237**
	LLR(–1)	0.005437***	0.005624***	0.007545***	0.006089***	0.010221*
	AD	–0.025021***	–0.023374**	–0.021292***	–0.020107***	–0.014453**
	AD (–1)	–0.007479*	–0.006513**	–0.009111*	–0.006864*	–0.003248*
	WB	0.120745***	0.139758***	0.118128***	0.117424***	0.136539***
	WB^2	–0.09235***	–0.093120***	–0.074556***	–0.077791***	–0.091423***
	LogALS	–0.016735***	–0.014470***	–0.040313***	–0.040173***	–0.036871**
	ΔLogALS	–0.056812***	–0.058473***	–0.040592***	–0.034812***	–0.039181***
	LogGINI/CAPITA	0.017638**	0.029767***	–0.017150*	–0.009892	–0.038173***
	LogGINI/CAPITA (–1)	0.017638**	0.020519***	0.034077***	0.038025***	0.034199***
	LogALP	0.046365	0.001813*	0.012315*	–0.002735	0.036278***
	LogALP^2	–0.002748	0.002853***	0.012417*	0.001116	0.035167***
External factors	INFL	0.155227***	0.192117***	0.083014***	0.084629***	0.126562***
	ΔINFL	0.029297	0.092728***	0.058234	0.044564	0.126463***
	COMP	–0.000350***	–0.000332***	–0.00208	–0.000186***	–0.000134**
	REG	0.008089**	0.010330***	0.012429***	0.016493***	0.021724***
Interaction	LogALS*OC	–		–	0.215647**	0.203318***
	PR*OC	–		0.251875***	0.278657**	0.268031***
Constant R^2 Prob J-static SCE Instruments Observation			–0.088882 0.6865 0.000000 58.47333 27 5,075	–0.142721 0.605919 0.000000 73.32150 28 5075	–0.024847 0.605586 0.000000 73.38336 30 5,075	–0.028468 0.615144 0.000000 71.60500 21 5,075

***Significativity at 1% level; **Significativity at a 5% level; *Significativity at 10%.

In the second model, all non-significant variables were removed from the model. In the third model, we have integrated in addition to significant variables, the interaction between operating costs and profit. This is to observe if the impact of this latter on the interest rate can be better explained by the operating costs, all other things being equal. The fourth model incorporates the second level of interaction between the ALS and operational costs (OC). The last model considers only significant variables and different interactions.

### Interpretation of internal factor results

The estimation results from the different models show that the coefficients associated with the lagged real interest rate have a significantly positive relationship at 1%. This shows that MFIs’ interest rate rises can be persistent over time and that the anticipation phenomenon of these interest rates remains a reality in the microfinance sector. The money that remains capitalized for a longer time in an uncertain future, and the lower risk level in the short term, can explain this result.

Taking account of the anticipations allows MFIs to adjust the yield/risk relationship, in comparing the existing risk portfolios and the various assets. The consequence is that a short-term rise in interest rates affects long-term rates more quickly and more often. The consistency of results is due to the interest level at t period which depends on the interest level in t-1 past period.

In Table 10, we remark also that the coefficients of operational costs (OC) and that associated with the absolute variance of this variable are statistically significant. On this basis, it can be said that a short-term change in operational costs led to an increase in the MFI interest rate. In setting the interest rate, MFIs take into account current operating costs and changes in those costs between two periods. From this point of view Cuéllar-Fernández et al. (2016), specify that operational costs (OC) result in higher interest rates (Cuéllar-Fernández et al., 2016). They also argue that excluding this variable in the estimates may cause a considerable reduction in the global significatively of the model (i.e., generating a 31.6% decrease in R squared). Multiple reasons justify this operational cost impact on the interest rate. MFIs are common on the significant influence of some factors, which affect operational costs such as the loan amount, the geographical location, the density of the customers, and the loan types. The MFIs development generates operating costs that absorb almost all the incomes of the microcredit portfolios. For this reason, operating costs are a determining factor in interest rate fluctuations.

**TABLE 10:** Arellano-bond serial correlation test.

Test order	m-Statistic	Rho	SE (rho)	Prob.
AR(1)	–3.4944	–225.2819	64.4687	0.0005
AR(2)	–0.9798	–62.8970	64.1885	0.3271

Except for the operating costs, in the study, have the financial costs (FC) as an influential variable in the MFI interest rate. It is noted that a significant part of the MFI portfolio is funded by the financial debts on which MFIs pay interest. These resource costs represent the interest paid on the incurred financial debts and the interest paid on the deposits. The estimation results of the different models (I to V) show positive and significant (at the 1% level) coefficients associated with the variable FC and ΔFC. In other words, an increase in the current financial cost value, and a variation of this over time generates an interest rate increase.

MFIs anticipate, among other things, the increase in financial costs to determine their interest rate. The positive impact of the current financial cost value is confirmed in Cotler and Almazan (2013) studies, which have shown that this variable is a fundamental element in the definition of MFI yields. This phenomenon may be explained by MFIs development, which means that potential lenders tend to reduce their loan parts. Fact due to this, MFIs will increasingly turn to almost commercial borrowers at expensive lending rates on national or international financial markets. this is adding to the regulation effect, which makes lenders tend to turn to regulated MFIs.

The study also highlighted in these models the loan lost rate (LLR) as well as its lagged value. They represent bad debts or credits, which have delays against their deadline. Most of the time they are considered loans in default or restructured credits. This variable provides information on MFI interest rate fluctuations. Through the different models, we identify the coefficients associated with the current value of (LLR) variable and the lagged (LLR (–1) variables that have positive relationships. The interest rate increase is considered boosted by the loan lost rate (LLR) anticipation and its current value.

The sensitivity of this variable to MFI portfolios and the quality of risk portfolios may explain this impact. If this risk level is high, the MFIs should constitute reserves and therefore increase their interest rate to keep the MFI financial equilibrium.

Regarding female borrowers (FEMEMP), we note that it represents a determinant element in the interest rate MFI variation due to their reactivity and their repayment capacity. It is, therefore, a target for most MFIs operating in rural areas. MFIs bill them significant interest rates to offset loan lost credit they may incur with other borrowers. Our results show that an increase in female borrowers to the 67% threshold is accompanied by a real interest rate increase.

This threshold represents the ratio between the marginal effects of the two variables (β (for the woman)/2 ×β (for Woman^2). If the range of female borrowers is between 0 and 67% levels, the interest rate tends to increase by about 0.11 points (Model III, IV). Beyond this 67% threshold, the effect of this variable on the interest rate is decreasing. In other words, when women borrowers exceed this threshold, MFIs lower their interest rates. Other authors consider that the MFIs that serve a high female borrowers section are more likely to lower their real interest rate because women are more likely to repay (Pitt and Khandker, 1998). Brau and Woller (2004) conclude that there is an ambiguous relationship between women borrowers and interest rates (Brau and Woller, 2004). Net profit (PR) is also an important factor for MFIs. It allows them to be financially independent, to be developed without subvention granted, to access financial resources, and to reinvest. The results from the various estimates show a significant but negative association between the interest rate and the current profit (K) values. It is also noted that when the interaction between operational costs (OC) and the net profit rate (PR) is being taken into account in the estimations models, MFIs anticipate an increase in profit, explained by the operational costs decrease, which will reduce the interest rate. This shows that the anticipation phenomenon of the profit impact on the interest rate is better explained by the variety of operational costs. This result is different from other field studies whose perspective is that MFIs should not practice social missions if this comes as a disadvantage to their activity. Other authors argue that generating profits will only consolidate innovation in the sector.

Another variable that requires particular attention is customer deposits (AD). Our results show that the higher the deposits then the lower the MFI interest rates. The regression outcomes displayed coefficients associated with the current deposits which are negative and significant at a 1% level, as the anticipated deposit values are also significant. This phenomenon can be expressed through competition in the opening of bank accounts, which are almost free in the microfinance sector compared to those offered by conventional banks, which motivates most customers to turn to MFIs.

Customer deposits also provide MFI managers with a perspective situation of the lending portfolio at a specific time. In the short term, the deposit variation (AD) generates anticipation of MFI interest rates decreasing.

The results also showed that the ALS is a determinant factor in the fluctuations of MFI interest rates. The coefficients associated with the current variable (LogALS) and its absolute value changing (ΔLogALS) are significantly negative at 1% in all the estimated models (I–IV). An increase in this variable as well as a variation in this latter in the short-term causes an MFI interest rate to decrease. This means, that the IMF is very active. The outcomes have consistency with the results of Dorfleitner et al. (2013). This variable also reflects the MFI’s financial sustainability and performance level.

The interaction effect between the ALS (locals) and operational costs shows statistically significant outcomes. The negative impact of the ALS on the interest rate is partly explained by the operational costs, all other things being equal.

### Interpretation of the result of the external factors

External factors represent those variations that may have an indirect, positive, or negative impact on MFI interest rate variations and on which the MFI does not necessarily have the power to control. The external factors considered in this study are the market structure (Competition), economics (inflation), cultural and technological political conditions, and banking regulations in effect (regulation).

Inflation is an important external factor influencing MFIs. In the context of inflation rising, MFIs are often faced with an arbitration problem between a negative real interest rate that would negatively affect their loan portfolio quality as customers, save less, or negatively affect a nominal interest rate that covers inflation. In the first case, the objective of the IMF is to lower inflation, which will, therefore, boost the decreased purchasing power of households and thus their income. Whereas in the second case, the increase in the interest rate aims to contain inflation at a reasonable level, this would mechanically increase the household purchasing power by emphasizing the income increase.

This could encourage household savings with MFIs. The results express the significant positive relationship at the 1% level between current inflation and the interest rate level. In other words, an increase in inflation can cause an increase in the MFI interest rate. However, this relationship between interest rates and the absolute value changing in inflation is not significant but always positive. These results show that MFIs do not anticipate inflation in setting their interest rates.

Regulation (REG) is also an important factor in the microfinance sector for different reasons: it limits the MFI’s power over clients and minimizes the risk level of clients.

Thus, through the results provided in Table 5, the coefficient of variables is significantly positive at the level of 5%. This shows that regulation does not necessarily provide an interest rate decrease even if a regulated MFI is more likely to decrease its interest rate. This impact of the regulation on MFI interest rates may result from the restraint measures introduced by the government such as the interest rate ceiling, supervision or control, the requirement for minimum reserves, and restriction on some financial services that require additional costs for the MFI.

Regulation can also cause a changeover from a non-lucrative MFI to a lucrative MFI. As was the case in Bolivia in the 1990s, where the legislation introduced allowed a reduction of the financial activities of NGOs by obligating them to transform themselves into a joint-stock company to own a license. Facing a growing MFIdiversity, some believe that regulations should not be applied to all MFI types (CGAP, 2000)^1^. Therefore, our results are contradictory to those found by Dorfleitner et al. (2013) and Kacem and Zouari (2013) who have shown that regulation causes low rates of interest (Dorfleitner et al., 2013; Kacem and Zouari, 2013).

Competition (COMP) is an important instrument for innovation. Based on our results (Table 5) an increase of one unit in the competition leads to a significant decrease in the real interest rate (Model I to Model V). All coefficients associated with this variable are significantly negative. Thus, this phenomenon can be justified by the necessity for MFIs to provide quality services through advertising, and procedures for change in lending to get closer to and retain their target group.

### Analysis of the legal status effect

The legal status represents the legal entity of the microfinance institution, which allows for the management of all the rules structuring the institution’s activities. The different results obtained (Table 5) show how the different factors that are taken into account affect the MFI interest rate. Nevertheless, despite the relevance of these results, they do not specifically prove how these variables affect the interest rates defined within each legal status (NGO, NFIB, Rural Bank, Credit Union, Bank). In the existing literature, some authors (Dorfleitner et al., 2013) have developed the influence of legal status on interest rate evolution. Most of the research focuses on the relationship between financial health and legal status (Tchakoute-Tchuigoua, 2010).

To address this issue, the study presents an economic model considering the dynamic panel dimension by using the Fisher F-test which provides more correct results for such qualitative variables. The procedure is as follows:

Firstly, the unconstrained models are estimated. The sum of the squares errors noted (SE) is calculated.

The object is to test the hypothesis that legal status does not influence interest rate fluctuations, this hypothesis is written as:


$$\begin{array}{c} H_{0}:\beta \,\left(C\,U\right)=\beta \,\left(B\,A\,N\,Q\right)=\beta \,\left(R\,B\right)=\beta \,\left(N\,G\,O\right)=0 \end{array}$$

$$\begin{array}{c} H_{1}:\beta \,\left(C\,U\right)\neq \beta \,\left(B\,A\,N\,Q\right)\neq \beta \,\left(R\,B\right)\neq \beta \,\left(N\,G\,O\right)\neq 0 \end{array}$$

Secondly, under the null hypothesis H0 the models become constrained models, these models are estimated without the cooperative union (CU), Bank (BANQ), NGO, and rural banks (RB). We calculate the sum of the Error Sum of Scales (SSE).

The following statistic is used:


(4) $$F=\frac{\left(S\,S\,E_{c}-S\,S\,E\right)/m}{S\,S\,E/N-k}∼F_{m,N-k}$$

m shows constraints here, N is the total observations, while k is the coefficients of regression. This statistic follows Fisher’s law at m and n-k degrees of freedom.

The summary of outcomes (constrained and non-constrained models) are shown in the following table:

Table 11 shows Fisher’s and different statistics on legal status. Their calculation is based on the constrained and non-constrained models set out in the appendices. We notice that the different statistics generated are statistically significant. The coefficients associated with the different variables (CU, BANQ, RB, NGO) are significantly different from zero. Therefore, legal status provides a crucial role in the MFI interest rate fluctuations. To improve comprehension of the effect of legal status on the interest rate, we have studied in Table 12 how the explanatory variables affect the interest rates set by each legal status. This allows an evaluation of the MFI characteristics and vision of the similarities and differences between the MFI types to detect those that apply higher interest rates.

**TABLE 11:** Fisher statistics for the legal status.

Legal status effect on interest rate according to the Fisher test					

	Model I NIR	Model II NIR	Model III NIR	Model IV NIR	Modelé V NIR
SSEc	58,90707	59,20827	62,0451	58,05298	69,71664
SSE	57,82535	57,93503	60,56801	56,96197	67,54259
M	4,00	4,00	4,00	4,00	4,00
N	5,075	5,075	5,075	5,075	5,075
K	33	28,00	28,00	30,00	21,00
(SSEc-SSE)/m (1)	0,27	0,32	0,37	0,27	0,54
SS/(n-k) (2)	0,01	0,01	0,01	0,01	0,01
F = (1)/(2)	23,64523656	27,80643956	30,85602651	24,22414284	40,78199178
Conclusion I	***	***	***	***	***

Legal status effect on interest rate according to the Fisher test. *, **, and *** are significant at the confidence levels of 10%, 5%, and 1%, respectively.

**TABLE 12:** Interest rate determinant according to legal status.

	BanK	NFIB	NGO	UC	RB
					
Variables	Model II NIR	Model III NIR	Model IV NIR	Model VI NIR	Model VI NIR
NIR (–1)	0.066352***	0.042105***	0.054347***	0.027785	0.519187**
OC	0.879243***	0.929493***	0.816871***	2.012019***	1.009868***
FC	2.265633***	2.857378***	2.038515***	0.877564***	2.000751***
WOR	0.600460***	1.089773***	0.634035***	0.770931***	1.290096***
LLR	0.962133***	0.263215**	0.943239***	0.021094	–0.806765***
WB	–0.017172	0.014613	0.059544	0.003334	0.026802*
LOGALP	0.093869*	–0.010088***	–0.022317	0.008222***	–0.017236
LOGALS	–0.019851	–0.002768***	–0.003508*	–0.005129**	0.053770
LOGGNI/CAPITA	0.000355	–0.001802	0.003452	0.005001**	0.011465
LOGAD	–0.000874	–0.015413**	–0.000269	0.003361	0.030030*
PAR-30	0.119439**	0.031617**	–0.005450	0.018487	0.013322
PR	0.817425***	0.869121***	0.797787***	0.902600***	0.267513
REG	0.018613	–0.017450***	0.007936**	0.001786	–0.003247*
INFL	–0.003699	0.094061	0.119912***	–0.009860	0.075355
UC	–	–	–	–	–
NGO	–	–	–	–	–
BR BANQ	–	–	–	–	–
LOALP^2	–0.001490	0.001912*	0.000824*	–	0.000937
LOGALS^2	0.001000	0.002754***	–	–	–0.004164
WB*DO	–0.058970	–0.515439***	–0.004215	–	–
Constante	–0.930360**	0.124865***	0.165573	–V0.133807***	–0.239982
R-squared	0.939518	0.912884	0.908512	0.952124	0.979208
Adjusted R-squared	0.936302	0.911739	0.907322	0.949880	0.972087
J-statistic	0.000000	0.000000	0.085923	0.000000	0.000000
Mean dependent var	0.470618	0.479531	0.447345	0.309136	0.424727
Sum squared resid	1.776008	14.39128	6.558445	0.454919	0.028647
Instrument rank	27	28	26	25	26
Observations	517	2,006	1,872	538	100

*, **, and *** are significant at the confidence levels of 10%, 5%, and 1%, respectively.

Microfinance institutions can differentiate themselves according to their size, age, legal status, etc. In Table 7, the study highlights the variables to prove how this interest rate is defined according to the legal status chosen by the IMF. From this table and for each MFI type, we remark that the interest rate in the t-year is defined according to that for the t–1 year. The different coefficients associated with this variable and according to each legal status, except the credit unions (CU), are positive and significant at the 1% level. Otherwise, NGOs, NBFI, rural banks (RB), and banks (BANQ) generally anticipate an interest rate increase. This phenomenon of anticipation is more important in rural banks.

The results provided in this table demonstrate that the effect of financial costs (FC) on the interest rate determination is positive for all legal statuses. In other words, this variable causes a rise in interest. It is more important for NBFI and BANQ with significant coefficients at the 1% level (2.857378^***^, 2.26^***^, respectively). As for operational cost (OC), their impact on the interest rate increase is more important for credit unions (CU) and rural banks (RB) with a significantly positive at the level of 1%.

This can be explained by the small loans focused on by these MFI types. Based on these observations, we support that the applied interest rate by credit unions (CU) is lower than other MFI types and those applied by NBFI are higher. This is in line with Fernandes et al. (2016) who argue that the CU makes group loans, a solution that boosts cost minimization.

Analysis of other variables like women borrowers (WB), regulation (REG), and ALS provides different results depending on legal status. If we take into account the regulation, we notice a significant but negative impact on the rate of interest change for NBFI. This result proves the variance in interest rates between regulated and non-regulated NBFI. For the other legal statutes, except NGO, the various coefficients associated with regulated MFIs are positive and not significant.

According to these results, it can be said that there is a variance in interest rates between regulated and non-regulated NGOs. For women borrowers, the positive expected signs of the coefficients are obtained for all MFI types except the banks (BANQ). However, they remain non-significant for most, except for rural banks (BR) where a 1% increase in female borrowers generates a positive and significant interest rate fluctuation. The same pattern is noted for LLR and provisions where the coefficients are positive and significant for all MFI types except for rural banks (BR). The impact of LLRs on the interest rate is higher for BANQ and NGOs with a considerable increase in the LLR (Brihaye et al., 2019). As for provisions, they are more significant for the NBFI and the RB with respective coefficients of 1.08^***^ and 1.29^***^. The coefficients associated with PAR_30 for various legal statuses are positively significant for banks (BANQ) and financial institutions (NBFI). Indeed, a 1% increase in PAR_30 causes a 12% increase in bank interest rates and 3% for NBFI.

In summary, the general pattern provided by the estimates in Table 7 proves that the NBFI applies higher interest rates given that all the tested variables are significant for this legal status.

These different conclusions, extracted from the legal status effects on the determination of the interest rates, allow validation of the hypothesis that the interest rates of microfinance institutions also depend on the IMF’s legal status.

## Conclusion

The main objective of this paper was to study the determinant factors of the rate of interest fluctuations of microfinance enterprises based on the study limitations of Dorfleitner et al. (2013) and Rosenberg et al.’s (2013) research. For this purpose, we have used a dynamic panel analysis to understand the behavior of MFIs in interest rate settings.

MFIs anticipate these interest rate changes because the latter represent sensitive data to the slightest economic variations. Financial costs, operational costs, the write-off rate, and the average size of the loan are the most important determinant factors in MFI interest rate fluctuations. The outcomes of this research also show that MFIs set their interest rates above the parameters taking into account the Rosenberg et al. (2013) formula.

The study find that other factors like gender (number of female borrowers), legal status, and regulations also contribute to the MFI interest rate variation. We have also discovered that there is a threshold effect in the relationship between women borrowers (WB) and the interest rate. In other words, women pay higher interest rates if their number in the institution is below 68% and low-interest rates if the percentage of women borrowers in the MFI exceeds this threshold. According to the legal status of the institution, we have demonstrated that NBFI clients bear higher interest rates compared to other MFIs. Another important finding of this study is that MFIs do not anticipate inflation in the definition of the interest rate. Rather, they incorporate current inflation to define the interest rate, which is a recurring phenomenon in developing countries.

Based on our study, a few key propositions emerge that, if applied, would likely keep the MFI interest rate at a reasonable and fair level. It is important to find ways to streamline operational and financial costs. Financial alphabetization must also be promoted to enable consumers to understand the MFI organization. From an institutional point of view, it is necessary to promote competition, as the study shows that well-regulated competition helps to keep interest rates at a reasonable level.

This study has a substantial field contribution and the relevance of the results is widespread to all MFIs in the world with a representative sample size (5075 observations) and relevant variables.

The methodological approach through the dynamic method used in this paper is novel and is not used in the microfinance institution literature before this study. Nevertheless, despite these important conclusions, some limits are worth noting. This paper can better be explained by distinguishing regulated and unregulated MFIs in the course of the research. Research can be also expanded on the borrowers to have a double facet of the MFI interest rate as they have a role in MFI interest rate determination and because they change their attitude against interest rate variation.

## Data availability statement

The datasets presented in this study can be found in online repositories. The names of the repository/repositories and accession number(s) can be found in the article/supplementary material.

## Ethics statement

Ethical review and approval was not required for the study on human participants in accordance with the local legislation and institutional requirements. Written informed consent from the participants’ legal guardian/next of kin was not required to participate in this study in accordance with the national legislation and the institutional requirements.

## Author contributions

FR and WW: conceptualization and data curation. WW: methodology and supervision. FR: software, formal analysis, investigation resources, and visualization. FR, WW, LJ, SR, SQ, and KN: validation, writing—original draft preparation, and review and editing. FR and LJ: project administration. All authors have read and agreed to the published version of the manuscript.

## Footnotes

1 CGAP is a donor coordinator in microfinance and a disseminator of microfinance best practices to policymakers and practitioners (see www.cgap.org).
